# GABA-A Receptor Modulation and Anticonvulsant, Anxiolytic, and Antidepressant Activities of Constituents from *Artemisia indica* Linn

**DOI:** 10.1155/2016/1215393

**Published:** 2016-04-07

**Authors:** Imran Khan, Nasiara Karim, Waqar Ahmad, Abeer Abdelhalim, Mary Chebib

**Affiliations:** ^1^Department of Pharmacy, University of Swabi, Khyber Pakhtunkhwa 23340, Pakistan; ^2^Department of Pharmacy, University of Malakand, Khyber Pakhtunkhwa 18800, Pakistan; ^3^Faculty of Science, Taibah University, Medina 30001, Saudi Arabia; ^4^Faculty of Pharmacy, University of Sydney, Sydney, NSW 2006, Australia

## Abstract

*Artemisia indica*, also known as “Mugwort,” has been widely used in traditional medicines. However, few studies have investigated the effects of nonvolatile components of* Artemisia indica* on central nervous system's function. Fractionation of* Artemisia indica* led to the isolation of carnosol, ursolic acid, and oleanolic acid which were evaluated for their effects on GABA-A receptors in electrophysiological studies in* Xenopus* oocytes and were subsequently investigated in mouse models of acute toxicity, convulsions (pentylenetetrazole induced seizures), depression (tail suspension and forced swim tests), and anxiety (elevated plus maze and light/dark box paradigms). Carnosol, ursolic acid, and oleanolic acid were found to be positive modulators of *α*1*β*2*γ*2L GABA-A receptors and the modulation was antagonized by flumazenil. Carnosol, ursolic acid, and oleanolic acid were found to be devoid of any signs of acute toxicity (50–200 mg/kg) but elicited anticonvulsant, antidepressant, and anxiolytic activities. Thus carnosol, ursolic acid, and oleanolic acid demonstrated CNS activity in mouse models of anticonvulsant, antidepressant, and anxiolysis. The anxiolytic activity of all three compounds was ameliorated by flumazenil suggesting a mode of action via the benzodiazepine binding site of GABA-A receptors.

## 1. Introduction

*Artemisia indica* is known as “Mugwort” in the local community in South Asia and belongs to the family “Asteraceae.” The plant is perennial shrub with height of 2–8 meters tall, found in Northern area of Pakistan as well as in Asia and Europe at high altitude of cold temperate zone [1].* Artemisia* species possesses antiplasmodial [2], antispasmodic and bronchodilator [3], antihypertensive [4], antiallergic [5], hepatoprotective [3], and antibacterial activities [6]. Furthermore, various species of this plant have been reported to possess antihyperglycemic effects as well [1]. Several species of* Artemisia* have been reported to possess CNS activities. For example,* Artemisia absinthium* has been shown to possess antidepressant activity in mouse models of tail suspension and forced swimming tests [7]. Also,* Artemisia dracunculus* has been reported to possess central antinociceptive effect [8].

Phytochemical studies have featured the presence of various classes of substances including essential oils, flavonoids, amino acids, triterpenes, coumarins, phenolic compounds, and carotenoids [9, 10]. The flavonoids isolated from various species of Asteraceae include apigenin-4-O-methyl ether, isoscutellarein-8-O-methyl ether, isoscutellarein-8,4-dimethyl ether, 8-methoxyluteolin, 5,3,4-trihydroxy-6,7,8-trimethoxyflavone, 5,7,3,4-tetrahydroxy-3,6,8-trimethoxyflavone, and 5,3,4-trihydroxy-3,6,7,8-tetramethoxyflavone [11]. Terpenes isolated from various species of Asteraceae include monoterpenoids, triterpenoids, and sesquiterpenoids such as dihydroartemisinic acid, artemisinic acid, artemisinin, and arteannuin B [12]. Various phenolic compounds isolated from Asteraceae family include caffeoyl derivatives, that is, chlorogenic acid, cichoric acid, 1,5-, 3,5-, and 4,5-di-*O*-caffeoylquinic acids, and 3-caffeoylquinic acid [13]. Chlorogenic acid isolated from* Artemisia capillaris* has been shown to possess antidepressant activity [14]. Flavonoids from* Artemisia herba-alba* have been shown to possess in vitro GABA-A benzodiazepine receptor activity [15].

*γ*-Aminobutyric acid (GABA) is the major inhibitory neurotransmitter in the mammalian central nervous system (CNS) activating neurons through a number of pharmacologically and structurally different receptor subtypes: ionotropic GABA-A and GABA-C and metabotropic GABA-B receptors. GABA-A receptor plays an important role in the overall balance between neuronal inhibition and excitation [16]. The GABA-A receptor is possibly the most complex member of the ligand-gated ion channel and is a pharmacological target not only for GABA but also for general anaesthetics, barbiturates, picrotoxin, and neurosteroids and a number of flavonoids modulate GABA-A receptors in CNS [17, 18].

**Figure 1:**
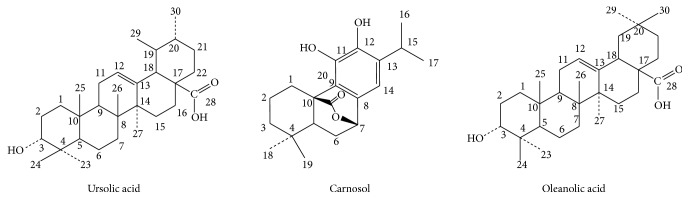
Chemical structure of the compounds isolated from* Artemisia indica* Linn.

Although the phytochemical profile of various* Artemisia* species has been extensively studied, this is the first study reporting the phytochemical and CNS pharmacological profile of* Artemisia indica*. Carnosol, ursolic acid, and oleanolic acid (Figure 1) isolated from* Artemisia indica* were evaluated for their positive modulatory effects on GABA-A receptors expressed in* Xenopus* oocytes and were subsequently investigated in mouse models of toxicity and anticonvulsant, antidepressant, and anxiolytic activity to correlate the in vitro and in vivo findings.

## 2. Materials and Methods

### 2.1. Chemicals, Materials, Instrumentation, and Drugs

All chemicals used were purchased from Aldrich Chemical Co., Ltd. (St. Louis, MO, USA), and were of highest commercially available purity. Silica gel for column chromatography (CC) was performed on silica gel (Merck silica gel 60H, particle size 5–40 *μ*m) and Sephadex LH-20 gel. Thin layer chromatography (TLC) was performed on Merck aluminium backed plates, precoated with silica (0.2 mm, 60F254). UV spectra were recorded on Hitachi's U-2000 Double-Beam UV/Vis Spectrophotometer. Mass spectra were carried out on a Thermo Finnigan PolarisQ Ion Trap system (Waltham, MA, USA) using a direct exposure probe. Nuclear magnetic resonance ^1^H NMR and ^13^C NMR spectra were recorded at 400 and 100 MHz, respectively, on a Varian Gemini spectrometer (Palo Alto, CA, USA). Melting points were determined using a Stuart SMP10 melting point apparatus (Stone, Staffordshire, UK).

Diazepam was kindly donated by the Department of Pharmacy, University of Peshawar. Imipramine, DMSO, Tween solution (TWEEN® 80), flumazenil (Sigma-Aldrich, USA), and pentylenetetrazol (Tokyo Chemical Industries Co., Ltd.) were purchased for the study. All chemicals and solvents used in this research were of analytical grade.

### 2.2. Plant Material and Extraction

The whole part of the plant was dried in the shade at room temperature and crushed into fine powder. 5 kg of dried powdered sample was extracted with 100% methanol. The methanol extract was filtered and three sample replicates were extracted under the same conditions with new solvent. The methanol extract was filtered and evaporated under reduced pressure using a vacuum rotary evaporator at 45°C which yields 700 g of semisolid crude methanolic extract of* Artemisia indica*. The crude methanolic extract was then suspended in deionized water and partitioned sequentially with n-hexane, chloroform, ethyl acetate (EtOAc), and butanol (water saturated BuOH) fractions and was then evaporated using a vacuum rotary evaporator to get different extracts.

### 2.3. Purification

In the preliminary assay of the crude methanolic extract of* Artemisia indica* Linn, the chloroform fraction was found to be the most active one and possessed various important pharmacological activities. The chloroform fraction 250 g was subjected to column chromatography (Φ28 × 4.5 cm) on silica gel (70–230 mesh size) for further isolation and purification which yielded the three new compounds from* Artemisia indica* Linn for the first time. These included ursolic acid, carnosol, and oleanolic acid.

The chloroform fraction (250 g) was subjected to column chromatography (Φ28 × 4.5 cm) which was initially eluted with pure hexane and then the polarity gradually increased using ethyl acetate affording three subfractions F1, F2, and F3. F1 (62 g) was further subjected to column chromatography (Φ24 × 3 cm) with 5% ethyl acetate/hexane as the solvent system which afforded carnosol (3 g). F2 (47.8 g) was subjected to column chromatography (Φ25 × 3 cm) (8% ethyl acetate/hexane as the solvent system) that gave the ursolic acid (3.8 g). F3 (obtained from the soluble fraction of 200 g of chloroform) was separated by column chromatography (Φ21 × 3.5 cm) to yield oleanolic acid (2 g) (15% ethyl acetate/hexane as the solvent system).

### 2.4. Spectroscopic Data of the Isolated Compounds

*Ursolic Acid*. Colorless needles; ESMS* m/z* (%): 479.4 (100), 457 (5), 439 (15), 425 (44), 413 (12). IR spectrum showed the presence of carbonyl at (1697 cm^−1^), OH group at absorption (3510 cm^−1^), and trisubstituted double bond at (1635 and 820 cm^−1^). The ^1^H NMR (DMSO-*d*_6_) *δ* ppm: 2.99 (^1^H, s, H-3), 5.11 (^1^H, s, H-12), 2.08 (^1^H, d, H-18), 0.89 (^1^H, s, H-23), 0.73 (^1^H, s, H-24), 0.65 (^1^H, s, H-25), 0.87 (^1^H, s, H-26), 1.02 (^1^H, s, H-27), 0.79 (^1^H, d, H-29), 0.84 (^1^H, d, H-30), 2.08 (^1^H, d, *J* = 11.6 Hz, H-28). ^13^C NMR (DMSO-*d*_6_) *δ* ppm: 38.7 (C-1), 27.4 (C-2), 77.3 (C-3), 38 (C-4), 55.2 (C-5), 18.4 (C-6), 33.2 (C-7), 39.5 (C-8), 47.5 (C-9), 37 (C-10), 23.3 (C-11), 125 (C-12), 138.6 (C-13), 42.1 (C-14), 28.7 (C-15), 24.3 (C-16), 47.3 (C-17), 52.8 (C-18), 38.9 (C-19), 38.8 (C-20), 30.6 (C-21), 36.8 (C-22), 28.7 (C-23), 16.5 (C-24), 15.70 (C-25), 17.4 (C-26), 23.7 (C-27), 178.7 (C-28), 17.5 (C-29), 21.5 (C-30). 

*Carnosol*. White solid; UV *λ*_max_: 209 and 285 nm; ESMS* m/z* (%): 683.4 (100), 331.0 (19); ^1^H NMR (DMSO-*d*_6_) *δ* ppm: 2.64dd, 2.43dd (H-*α*:*β*, H-1), 1.5 m (H-2), 1.42dd, 1.21dd (H-*α*:*β*, H-3), 1.59dd (H-5), 2.01ddd, 1.6 m (H-*α*:*β*, H-6), 5.44dd (H-7), 6.68s (H-14), 3.21s (H-15), 1.10d (H-16), 1.10d (H-17), 0.79s (H-18), 0.77s (H-19). ^13^C NMR (DMSO-*d*_6_) *δ* ppm: 29.2 (C-1), 19.0 (C-2), 41.0 (C-3), 34.6 (C-4), 45.4 (C-5), 29.7 (C-6), 77.4 (C-7), 132.0 (C-8), 122.3 (C-9), 48.3 (C-10), 143.7 (C-11), 143.5 (C-12), 134.7 (C-13), 111.7 (C-14), 26.6 (C-15), 23.1 (C-16), 23.2 (C-17), 31.2 (C-18), 19.8 (C-19), 175.9 (C-20). 

*Oleanolic Acid*. White solid; ESMS* m/z* (%): 456 (100), 431 (15), 391 (8); ^1^H NMR (DMSO-*d*_6_) *δ* ppm: 2.98 (^1^H, t, H-1), 5.14 (^1^H, H-12), 2.78 (^1^H, dd, H-18), 0.65 (^1^H, s, H-23), 0.85 (^1^H, s, H-24), 0.87 (^1^H, s, H-15). ^13^C NMR (DMSO-*d*_6_) *δ* ppm: 38.5 (C-1), 27.4 (C-2), 77.3 (C-3), 38.9 (C-4), 55.5 (C-5), 55.5 (C-6), 32.5 (C-7), 38.8 (C-8), 47.5 (C-9), 37.0 (C-10), 23.1 (C-11), 122.0 (C-12), 144.3 (C-13), 41.8 (C-14), 27.6 (C-15), 23.3 (C-16), 45.9 (C-17), 41.3 (C-18), 46.1 (C-19), 30.8 (C-20), 33.8 (C-21), 32.9 (C-22), 28.7 (C-23), 16.5 (C-24), 15.5 (C-25).

### 2.5. Pharmacological Activities

#### 2.5.1. Animals

Male Swiss mice with a weight range of 20 to 30 g were purchased from the National institute of health (NIH), Islamabad, Pakistan. Animals were housed in the Department of Pharmacy, University of Malakand's animal house, with fresh water and standard food available ad libitum. The animals were maintained at a 12-hour light and dark cycle and with room temperature of 22–25°C in the animal house. All animal procedures have been approved by the Departmental Animal Ethical Committee (DAEC/PHARM/2012/13) and were conducted according to the UK animal scientific procedure act, 1986.

### 2.6. GABA Receptor Subunit Constructs

Human GABA-A *α*1, *β*2, and *γ*2L cDNA subcloned in pCDM8 were linearized with the appropriate restriction endonucleases and capped transcripts were produced from linearized plasmids using the “mMessage mMachine” T7 transcript kit from Ambion (Austin, TX, USA). The quality of mRNA was determined by 0.5% agarose gel electrophoresis. mRNA concentrations were measured by* NanoDrop*® ND-1000 UV-Vis Spectrophotometer. mRNA was diluted with nuclease-free water and stored at −80°C.

### 2.7. Expression of Recombinant GABA Receptors in* Xenopus* Oocytes

#### 2.7.1. Oocyte Preparation

Sexually mature female* Xenopus laevis* were kept under optimal conditions by authorized animal keepers at the University of Sydney Animal House and fed standard frog food. Stages V-VI oocytes were harvested under 0.1% tricaine (3-aminobenzoic acid ethyl ester) anesthesia. Oocytes were defolliculated by shaking for approximately 1 h at 18°C in collagenase (2 mg/mL) dissolved in OR2 solution containing (in mmol/L) NaCl 96, KCl 2, CaCl_2_ 1, MgCl_2_ 1, and HEPES 5, pH 7.4.

Stages V-VI oocytes were sorted and injected (Nanoject, Drummond Scientific Co., Broomall, PA) with cRNA reconstituted in nuclease-free water in a ratio of 1*α* (25 ng) : 1*β* (25 ng) : 5 (*γ*) (125 ng). Oocytes were incubated for up to 4–8 days in standard ND96, pH 7.4, supplemented with pyruvate (2.5 mM), theophylline (0.5 mM), gentamycin (50 *μ*g/mL), and 2% horse serum at 18°C.

#### 2.7.2. Electrophysiology

Currents were recorded using the two-electrode voltage clamp technique as described elsewhere [19]. Glass microelectrodes were filled with 3 M KCl (0.5–2 MΩ). Oocytes were clamped at −60 mV and continuously superfused with ND96 solution (96 mM NaCl, 2 mM KCl, 1 mM MgCl_2_, 1.8 mM CaCl_2_, and 5 mM HEPES, pH 7.5). Current amplitudes were calculated off-line using Chart software v3.6 (ADInstruments, NSW, Australia). Responses to GABA applications were normalized as *I*% = (*I*/*I*_max_) × 100, where *I* is the peak amplitude of current response and *I*_max_ is the maximum current produced by GABA measured from individual cells.

Modulation of GABA-elicited currents was tested by coapplying increasing concentrations of the drugs with a concentration of GABA that produced 5% of maximal activation (EC_5_, determined for each cell). Current responses were recorded and normalized as % potentiation = ((*I*_drug+GABA_ − *I*_GABA_)/*I*_GABA_) × 100, where *I*_drug+GABA_ is the control GABA current in the presence of a given concentration of drug and *I*_GABA_ is the amplitude of the control GABA current alone.

### 2.8. Acute Toxicity

The acute toxicity study was conducted according to the modified method described by Adebiyi and Abatan [20]. Swiss albino mice (25–30 g) were randomly divided into ten groups (*n* = 6). Animals were fasted overnight but had access to water ad libitum and then were treated intraperitoneally with carnosol, ursolic acid, or oleanolic acid (50, 150, and 200 mg/kg). Mice in the control group received 10 mL/kg i.p. of vehicle (0.9% w/v). Animals were keenly observed for any signs of toxic effects during the first 6 hours after the treatment and then carefully monitored for the subsequent 66 hours (3-day observation) for any changes in behaviors including grooming, hyperactivity, sedation, respiratory arrest, convulsion, increased or decreased motor activity, and mortality, if any.

### 2.9. Assessment of Anxiolytic Activity

#### 2.9.1. Elevated Plus Maze (EPM)

The apparatus consisted of two open arms (30 cm × 5 cm) and two closed arms (30 cm × 5 cm × 15 cm) made of black plexiglas connected by an open central platform (5 cm × 5 cm) and elevated 40 cm from ground level. A raised ledge (3 mm high and 1 mm thick) surrounded the perimeter of the open arms. Mice were injected with vehicle or drug and 20 min later placed in the center of the apparatus facing an open arm and allowed to explore the maze for 5 min. An arm entry was defined by having all four paws inside the arm. All sessions were videotaped by a camera positioned above the maze, and, at the end of the test, the number of arm entries and time spent in arms were recorded. To assess the involvement of the GABAergic system, animals were given vehicle or flumazenil (2.5 mg/kg) i.p. 20 minutes before treatment with carnosol, ursolic acid, or oleanolic acid. The % of open-arm entries and the % of time spent in the open arms were recorded as a measure of anxiety state [18].

#### 2.9.2. Light/Dark Box Test (LDB)

The light/dark apparatus consists of an acrylic box of dimensions 44 cm × 21 cm × 21 cm, divided into a small dark compartment (one-third) and a large illuminated compartment (two-thirds); the division between zones contains an opening of 6 cm × 3 cm. The box is equipped with 16 light beams, 11 in the lit area and 5 in the dark area, which detect the movement of the animal. The box is connected to a computer that records the number of transitions between areas, latency to the first transition, time and activity in each zone, and total activity in a 5 min session. An increase in the exploration of the lit area is associated with an anxiolytic effect; as such, two parameters were selected as a measure of anxiety: the time spent in the lit compartment and the total number of transitions [18].

### 2.10. Assessment of Anticonvulsant Activity

#### 2.10.1. PTZ-Induced Seizures

PTZ-induced convulsions test was used to evaluate anticonvulsant activity of carnosol, ursolic acid, and oleanolic acid [18]. In this test, 48 mice were divided into eight groups of six animals each. Group I (control) was treated with vehicle (5% DMSO, 1% Tween 80, and 94% saline) i.p. Group II received standard drug diazepam i.p. in a dose of 1 mg/kg. Groups II, III, IV, and V were treated with carnosol; groups VI, VII, VIII, and IX were treated with ursolic acid, whereas groups X, XI, XII, and XIII were treated with oleanolic acid i.p. in a dose of 1, 10, 30, and 100 mg/kg, respectively. All the drugs were given thirty minutes before PTZ (70 mg/kg). Each animal was placed in a transparent chamber for observation lasting forty-five minutes after i.p. injection of PTZ. The onset of seizure, duration, and mortality in treated mice were recorded. Mice that did not convulse 60 min after PTZ administration were considered to be protected.

### 2.11. Assessment of Antidepressant Activity

#### 2.11.1. The Tail Suspension Test

Mice were hung by their tail on the tail hanger using sticky tape for tail fixation, at approximately 1 cm from the end of the tail. The hanger was fixed in the black plastic box (20 × 20 × 45 cm) with the opening at the top front. The distance between the hanger and floor was approximately 40 cm. The mouse was suspended in the air by its tail and the immobility time was recorded over a period of 5 minutes. The duration of immobility was defined as the absence of all movements except for those required for respiration [21].

#### 2.11.2. The Forced Swim Test

The forced swim test was carried out in a glass cylinder (diameter 20 cm, height 30 cm) filled with water to the height of 20 cm. The water temperature was approximately 25–28°C. Mice were gently placed into the water and the immobility time was recorded by an observer over a period of 5 minutes. Immobility was defined as absence of all movements and remaining floating passively in the water with the head just above the water surface [22].

### 2.12. Locomotor Activity

The locomotor activity arena measured 50 × 40 cm and the floor was divided by lines into 4 equal-sized rectangular zones. Mice (*n* = 6 per group) were habituated to laboratory conditions 90 min prior to testing. Doses of carnosol, ursolic acid, oleanolic acid, or vehicle were administered intraperitoneally and animals were placed in the recording apparatus 30 min later. Group mean line-crossing counts were subsequently recorded between 1 and 30 min following introduction to the arena by means of a Cat Eye camera, coupled to a remote personal computer [23].

### 2.13. Statistical Analysis

Data were expressed as mean ± SEM. When several treatments were compared, one-way ANOVA was used and post hoc comparisons between vehicle and drug treated groups were made using Dunnett's multiple comparison test using GraphPad Prism version 5. In all tests, the criterion for significance was *P* < 0.05.

## 3. Results

### 3.1. Positive Modulation of GABA-Induced Currents at *α*1*β*2*γ*2L GABA-A Receptors

**Figure 2:**
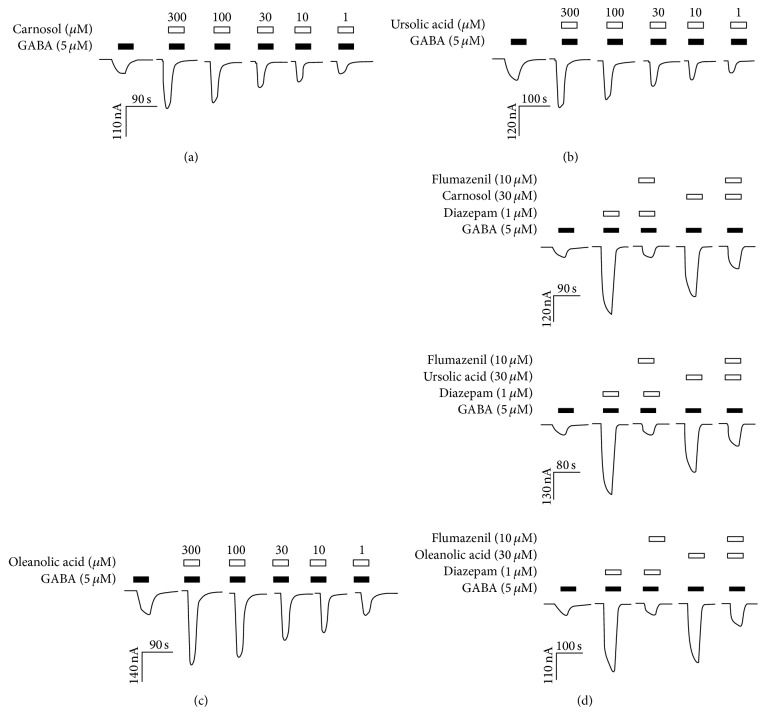
Carnosol, ursolic acid, and oleanolic acid are positive modulators of *α*1-containing GABA-A receptors expressed in* Xenopus* oocytes. Representative current trace showing the potentiation of GABA-EC5 by various concentrations of carnosol, ursolic acid, and oleanolic acid (1, 10, 30, 100, and 300 *μ*M) at human recombinant *α*1*β*2*γ*2L GABA-A receptors ((a)–(c)). The potentiating effect of carnosol, ursolic acid, and oleanolic acid was attenuated by flumazenil (10 *μ*M) similar to that of diazepam (d). Horizontal bars show the duration of drug application.

Carnosol, ursolic acid, and oleanolic acid enhanced the response to 5 *μ*M GABA at *α*1*β*2*γ*2L GABA-A receptors (Figure 2). Figures 2(a)–2(c) show the concentration dependent enhancement of GABA-EC5-evoked response by carnosol, ursolic acid, and oleanolic acid (1, 10, 30, 100, and 300 *μ*M) at *α*1*β*2*γ*2L receptors. Carnosol, ursolic acid, and oleanolic acid at 300 *μ*M maximally potentiated GABA-EC5-induced currents by 250 ± 30%, 230 ± 25%, and 220 ± 20% at *α*1*β*2*γ*2L receptors, respectively. We then studied the ability of neutralizing benzodiazepine antagonist flumazenil to block the potentiation of carnosol, ursolic acid, and oleanolic acid at *α*1*β*2*γ*2L GABA-A receptors. The positive modulation by carnosol, ursolic acid, and oleanolic acid at *α*1*β*2*γ*2L GABA-A receptors was mostly sensitive to antagonism by flumazenil (10 *μ*M) (Figure 2(d)). This effect was similar to the antagonism of diazepam induced potentiation of GABA effects at *α*1*β*2*γ*2L GABA-A receptors, indicating that these compounds bind to the benzodiazepine site of GABA-A receptors.

### 3.2. Acute Toxicity

Carnosol, ursolic acid, and oleanolic acid in the dose range of 50–200 mg/kg did not produce any noticeable effects on grooming, sedation, respiratory arrest, convulsions, or muscle activity. Furthermore, not a single case of lethality was found. Food and water consumption remained normal throughout the test period.

### 3.3. Assessment of Anxiolytic Activity in EPM

**Figure 3:**
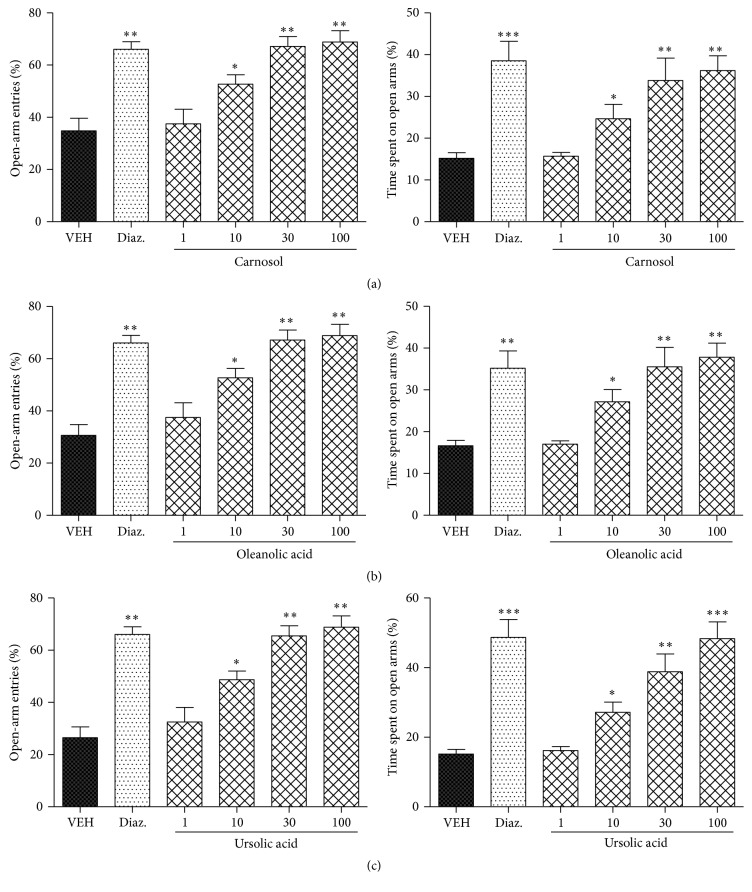
Effect of carnosol, oleanolic acid, ursolic acid, and diazepam on the behavior of mice in the elevated plus maze. (a) % of open-arm entries (left panel) and % of time spent in open arms (right panel) registered over a session of 5 min after 20 min of an i.p. injection of carnosol (1, 10, 30, and 100 mg/kg). (b) % of open-arm entries (left panel) and % of time spent in open arms (right panel) registered over a session of 5 min after 20 min of an i.p. injection of oleanolic acid (1, 10, 30, and 100 mg/kg), diazepam (1 mg/kg), or vehicle. (c) % of open-arm entries (left panel) and % of time spent in open arms (right panel) registered over a session of 5 min after 20 min of an i.p. injection of ursolic acid (1, 10, 30, and 100 mg/kg). Columns represent mean ± SEM (*n* = 6/group). ^*∗*^ *P* < 0.05, ^*∗∗*^ *P* < 0.01, and ^*∗∗∗*^ *P* < 0.001 compared with vehicle group using one-way ANOVA followed by Dunnett's test.

The anxiolytic effects of carnosol, ursolic acid, and oleanolic acid or diazepam in the elevated plus maze using diazepam as a positive control are summarized in Figure 3. Carnosol, ursolic acid, and oleanolic acid significantly reduced the anxiety in mice. All the three compounds at the doses of 10, 30, and 100 mg/kg i.p. significantly increased the % of open-arm entries (^*∗*^ *P* < 0.05 and ^*∗∗*^ *P* < 0.01; *n* = 6, one-way ANOVA followed by Dunnett's test) and % of time spent in open arms of the elevated plus maze (EPM) (^*∗*^ *P* < 0.05, ^*∗∗*^ *P* < 0.01, and ^*∗∗∗*^ *P* < 0.001; *n* = 6, one-way ANOVA followed by Dunnett's test).

Similarly, diazepam (1 mg/kg, i.p.) also increased % of open-arm entries (^*∗∗*^ *P* < 0.01) and the % of time spent in open arms of the EPM (^*∗∗∗*^ *P* < 0.001; *n* = 6, one-way ANOVA followed by Dunnett's test).

### 3.4. Effect of Flumazenil on the Anxiolytic Activity of Carnosol, Ursolic Acid, and Oleanolic Acid in the Elevated Plus Maze

**Figure 4:**
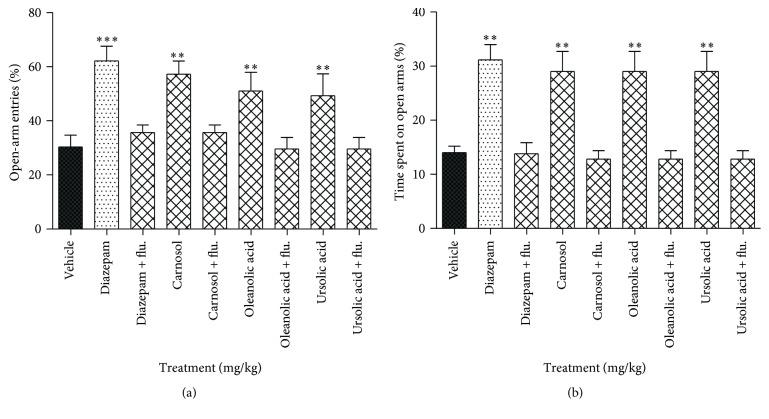
Effect of flumazenil on the anxiolytic activity of carnosol, oleanolic acid, and ursolic acid in the elevated plus maze. (a) % of open-arm entries and (b) % of time spent in open arms registered over a session of 5 min after 20 min of an i.p. injection of vehicle, diazepam (1 mg/kg), carnosol, oleanolic acid, and ursolic acid (10 mg/kg) in the presence of flumazenil (2.5 mg/kg). Columns express mean ± SEM (*n* = 6/group). ^*∗∗*^ *P* < 0.01 and ^*∗∗∗*^ *P* < 0.001 compared with vehicle control group using one-way ANOVA followed by Dunnett's test.

The anxiolytic activity of carnosol, oleanolic acid, and ursolic acid was decreased by coadministration of flumazenil (2.5 mg/kg) with carnosol, oleanolic acid, and ursolic acid (30 mg/kg), indicating that the benzodiazepine binding site of GABA-A receptors is involved in the anxiolytic activity induced by these compounds (Figure 4).

### 3.5. Assessment of Anxiolytic Activity in Light Dark Test

**Figure 5:**
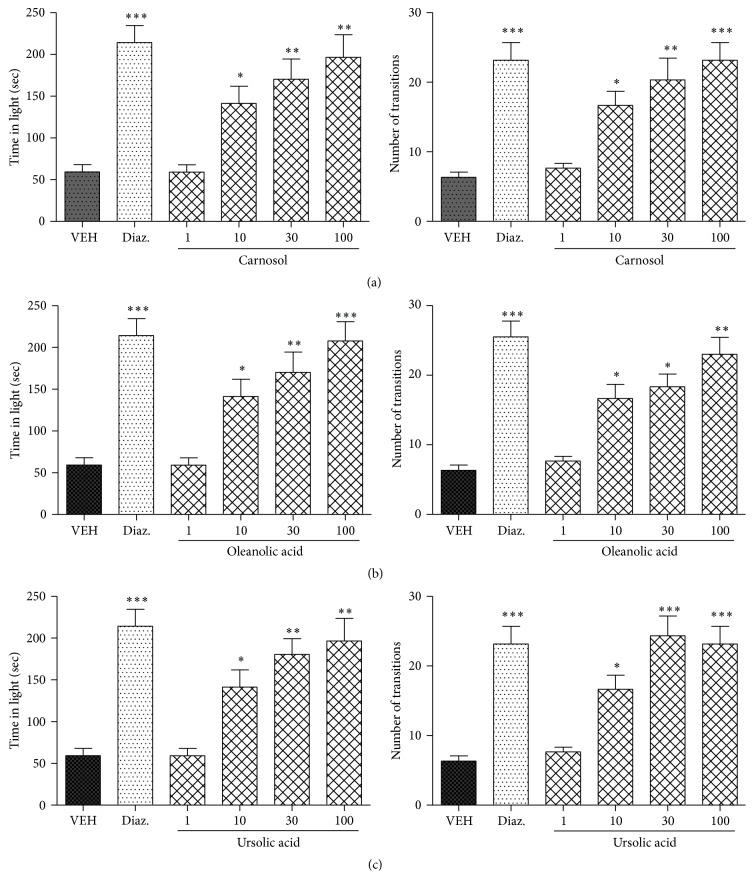
Effect of carnosol, oleanolic acid, ursolic acid, and diazepam on the behavior of mice in the light dark box test. (a) Time spent in lit area (left panel) and number of transitions (right panel), recorded over a session of 5 min, after 20 min of an i.p. injection of carnosol (1, 10, 30, and 100 mg/kg), diazepam (0.5, 1, and 3 mg/kg), or vehicle. (b) Time spent in lit area (left panel) and number of transitions (right panel), recorded over a session of 5 min, after 20 min of an i.p. injection of oleanolic acid (1, 10, 30, and 100 mg/kg), diazepam (0.5, 1, and 3 mg/kg), or vehicle. (c) Time spent in lit area (left panel) and number of transitions (right panel), recorded over a session of 5 min, after 20 min of an i.p. injection of ursolic acid (1, 10, 30, and 100 mg/kg), diazepam (0.5, 1, and 3 mg/kg), or vehicle. Values are expressed as mean ± SEM (*n* = 6/group). ^*∗*^ *P* < 0.05, ^*∗∗*^ *P* < 0.01, and ^*∗∗∗*^ *P* < 0.001 compared with vehicle group using one-way ANOVA followed by Dunnett's test.

The anxiolytic effects of carnosol, oleanolic acid, and ursolic acid in the light dark box test are shown in Figure 5. The results indicate that carnosol, oleanolic acid, and ursolic acid at the doses of 10, 30, and 100 mg/kg i.p. significantly increased the time spent in the light compartment (^*∗*^ *P* < 0.05, ^*∗∗*^ *P* < 0.01, and ^*∗∗∗*^ *P* < 0.001; *n* = 6, one-way ANOVA followed by Dunnett's test) and the number of transitions between light and dark compartments (^*∗*^ *P* < 0.05, ^*∗∗*^ *P* < 0.01, and ^*∗∗∗*^ *P* < 0.001; *n* = 6, one-way ANOVA followed by Dunnett's test).

The positive control drug diazepam (1 mg/kg, i.p.) also significantly (^*∗∗∗*^ *P* < 0.001) increased the time spent in the light area of the light dark box, indicating antianxiety activity and confirming that the paradigm is valid.

### 3.6. Assessment of Antidepressant Activity in Tail Suspension Test

The effects of carnosol, ursolic acid, and oleanolic acid on active behavior in the tail suspension test (TST) are shown in Table 1. Intraperitoneal administration of the positive control, imipramine, at the dose of 60 mg/kg, caused a significant reduction in the immobility time as compared to the vehicle (^*∗∗∗*^ *P* < 0.001). Carnosol, ursolic acid, and oleanolic acid at the doses of 10, 30, and 100 mg/kg caused a significant decrease in the immobility time as compared to the vehicle control group (^*∗*^ *P* < 0.05; ^*∗∗*^ *P* < 0.01).

### 3.7. Assessment of Antidepressant Activity in Forced Swim Test

The effects of carnosol, ursolic acid, and oleanolic acid on active behavior in the forced swim test (FST) of mice are summarized in Table 2. Intraperitoneal administration of carnosol, ursolic acid, and oleanolic acid at doses of 10, 30, and 100 mg/kg significantly decreased the immobility time in the FST as compared to the vehicle control (^*∗*^ *P* < 0.05; ^*∗∗*^ *P* < 0.01; ^*∗∗∗*^ *P* < 0.001; one-way ANOVA followed by Dunnett's test). The reference drug imipramine 60 mg/kg also caused a significant decrease in the immobility time as compared to vehicle control (^*∗∗∗*^ *P* < 0.001; one-way ANOVA followed by Dunnett's test).

### 3.8. Locomotor Activity of Carnosol, Ursolic Acid, and Oleanolic Acid

Keeping in view the possible effect of exploration on antidepressant effect, the effects of carnosol, ursolic acid, and oleanolic acid on locomotor activity were evaluated. The results showed that carnosol, ursolic acid, and oleanolic acid, at the dose level of 1–100 mg/kg, i.p., did not have any significant effect on locomotor activity compared to the vehicle (Table 3). At 100 mg/kg, all the three compounds caused a slight reduction in locomotor line crossing; however, this effect was statistically not significant.

### 3.9. Anticonvulsant Actions of Carnosol, Ursolic Acid, and Oleanolic Acid in PTZ Test

Carnosol, ursolic acid, and oleanolic acid showed significant anticonvulsant activity against PTZ-induced convulsions in mice. Carnosol, ursolic acid, and oleanolic acid caused a dose dependent increase in the duration of onset of tonic-clonic convulsions and decreased the duration of convulsions. Carnosol, ursolic acid, and oleanolic acid at doses of 10, 30, and 100 mg/kg caused a significant increase in the duration of onset (^*∗*^ *P* < 0.05; ^*∗∗*^ *P* < 0.01; ^*∗∗∗*^ *P* < 0.001; ANOVA with Dunnett's post hoc test; *n* = 6) and decreased the duration of clonic-tonic seizures (^*∗*^ *P* < 0.05; ^*∗∗*^ *P* < 0.01; ANOVA with Dunnett's post hoc test; *n* = 6). The reference anticonvulsant diazepam at doses of 1 mg/kg also significantly delayed the onset of clonic-tonic seizures and decreased the duration of clonic-tonic seizures (^*∗∗∗*^ *P* < 0.001; ANOVA with Dunnett's post hoc test; *n* = 8). Carnosol, ursolic acid, and oleanolic acid caused a dose dependent reduction in PTZ-induced mortality and provided 100, 83.33, 66.7, and 33.34% protection at doses of 100, 30, 10, and 1 mg/kg, respectively (Table 4).

## 4. Discussion

In the present study, we have evaluated three constituents, carnosol, ursolic acid, and oleanolic acid, from* Artemisia indica* for their ability to modulate GABA-induced currents at GABA-A *α*1*β*2*γ*2L receptor subtype expressed in* Xenopus* oocytes. Carnosol, ursolic acid, and oleanolic acid were subsequently evaluated for various neuropharmacological activities in mouse models. The results indicate that carnosol, ursolic acid, and oleanolic acid were positive modulators of *α*1*β*2*γ*2L recombinant GABA-A receptor. Furthermore, the potentiation at *α*1*β*2*γ*2L GABA-A receptor subtype was blocked by flumazenil under conditions, where flumazenil also inhibited diazepam induced enhancement, indicating that carnosol, ursolic acid, and oleanolic acid elicit potentiation via the benzodiazepine binding site. A fraction of the positive modulation produced by carnosol, ursolic acid, and oleanolic acid remained flumazenil insensitive indicating that a component of GABA-A receptor potentiation might be mediated by nonbenzodiazepine sites. Such a flumazenil independent positive modulation of GABA-A receptors has previously been reported with flavonoids [17, 18].

Anxiolytics are known to exert their pharmacological action by causing an increase in GABAergic neurotransmission in the brain [18, 22]. The elevated plus maze and light dark tests are routinely used for the assessment of anxiolytic activity of substances in rodents [24–26]. GABA-A receptors are involved in the anxiolytics effects of benzodiazepines [27]. Flavonoids have also been shown to mediate their anxiolytic effects via modulation of GABA-A receptors [25, 28]. Recently, we have shown that synthetic flavones such as 2′-methoxy-6-methylflavone [24], 3-hydroxy-2′-methoxy-6-methylflavone [25], and viscosine [18] exert their anxiolytic effects via modulation of GABA-A receptors. In this study, carnosol, ursolic acid, and oleanolic acid exerted significant anxiolytic effects at the doses of 10–100 mg/kg in both the elevated plus maze and light dark tests. Furthermore, the anxiolytic effect exerted by carnosol, ursolic acid, and oleanolic acid was antagonized by flumazenil indicating that the effect is mediated through the benzodiazepine site of GABA-A receptors.

The anticonvulsant activity of carnosol, ursolic acid, and oleanolic acid was determined using chemically induced (PTZ test) convulsions in mice. The results of the current study indicate that carnosol, ursolic acid, and oleanolic acid at the dose level of 10–100 mg/kg possess significant anticonvulsant activity in mice in PTZ-induced seizures which may elicit seizures by inhibiting GABAergic mechanisms [18, 29, 30]. GABA is the major inhibitory neurotransmitter and glutamic acid is the excitatory neurotransmitter in the brain. The inhibition of GABA and enhancement of the action of glutamate neurotransmitters are believed to be the underlying factors in epilepsy [31]. GABA-A receptor agonists as well as drugs which allosterically modulate GABA-A receptors are useful in convulsive seizures [32, 33]. The standard antiepileptic drug diazepam is believed to be mediating its anticonvulsant action by enhancing the action of GABA at GABA-A receptors [34]. Thus it is possible that the anticonvulsant effects shown by carnosol, ursolic acid, and oleanolic acid against PTZ-induced seizures may be due to the activation of GABAergic neurotransmission.

The forced swimming and tail suspension tests are well known models of depression sensitive to all classes of antidepressant including monoamine oxidase inhibitors, tricyclic antidepressants, and selective serotonin reuptake inhibitors [20, 35]. Both tests are widely used screening methods for antidepressants in mice [20, 21]. Carnosol, ursolic acid, and oleanolic acid exerted significant antidepressant effect as evidenced by the reduction of immobility time in both the tail suspension test and the forced swimming test. To avoid the possibility of false positive effect, these compounds were evaluated for their effects on locomotor activity tests. Carnosol, ursolic acid, and oleanolic acid did not exert any significant effect on the locomotor activity indicating that the decrease in immobility in the forced swimming and tail suspension tests is caused by an antidepressant-like effect rather than a locomotor-enhancing effect. Studies have shown that GABA-A receptors are involved in the pathophysiology of depression [36]. Patients suffering from depression have been shown to have deficient GABA levels as well as decreased cortical GABA-A receptors. Thus those substances which mimic the GABAergic system may have potential effects on depression [36]. Since carnosol, ursolic acid, and oleanolic acid potentiated GABA-induced currents at *α*1*β*2*γ*2L GABA-A receptor, it is possible that the antidepressant effects shown by carnosol, ursolic acid, and oleanolic acid may also be due to the activation of GABAergic mechanisms.

## 5. Conclusions

In conclusion, carnosol, ursolic acid, and oleanolic acid modulate *α*1*β*2*γ*2L GABA-A receptors via the benzodiazepine binding site. Furthermore, carnosol, ursolic acid, and oleanolic acid demonstrated significant anticonvulsant, anxiolytic, and antidepressant activities in mouse models of anticonvulsant, antidepressant, and anxiolysis. We have also shown that the anxiolytic activity is mediated by the GABAergic system. However, further studies are required to investigate possible mechanisms of action of these compounds in epilepsy and depression and to investigate these compounds on other GABA-A receptor subtypes.

## Figures and Tables

**Table 1:** Effect of carnosol, ursolic acid, and oleanolic acid on immobility period (sec) of mice using tail suspension test.

Group	Treatment	Dose (mg/kg)	Immobility time (sec)
1	Control	—	170 ± 10.5

2	Carnosol	10	139.4 ± 13.5^*∗*^
3		30	115.7 ± 11.3^*∗∗*^
4		100	100.5 ± 10.2^*∗∗*^

5	Ursolic acid	10	135.4 ± 14.8^*∗*^
6		30	115.3 ± 11.5^*∗∗*^
7		100	105.1 ± 10.4^*∗∗*^

8	Oleanolic acid	10	125.8 ± 5.2^*∗*^
9		30	115.3 ± 10.3^*∗∗*^
10		100	95.6 ± 15.1^*∗∗*^

11	Imipramine	60	60.2 ± 11.6^*∗∗∗*^

All values are expressed in mean ± SEM (*n* = 6). ^*∗*^ *P* < 0.05, ^*∗∗*^ *P* < 0.01, and ^*∗∗∗*^ *P* < 0.001 compared to the vehicle group. Differences between groups were analyzed by analysis of variance (one-way ANOVA) followed by Dunnett's test.

**Table 2:** Effect of carnosol, ursolic acid, and oleanolic acid on immobility period (sec) of mice using forced swimming test.

Group	Treatment	Dose (mg/kg)	Immobility time (sec)
1	Control	—	184.6 ± 13.5

2	Carnosol	10	149.4 ± 10.5^*∗*^
3		30	125.7 ± 12.3^*∗∗*^
4		100	80.5 ± 11.2^*∗∗∗*^

5	Ursolic acid	10	135.4 ± 10.5^*∗*^
6		30	120.3 ± 11.4^*∗∗*^
7		100	74.9 ± 10.5^*∗∗∗*^

8	Oleanolic acid	10	148.8 ± 10.2^*∗*^
9		30	125.3 ± 11.3^*∗∗*^
10		100	95.6 ± 14.1^*∗∗∗*^

11	Imipramine	60	70.2 ± 12.6^*∗∗∗*^

All values are expressed in mean ± SEM (*n* = 6). ^*∗*^ *P* < 0.05, ^*∗∗*^ *P* < 0.01, and ^*∗∗∗*^ *P* < 0.001 compared to the vehicle group. Differences between groups were analyzed by analysis of variance (one-way ANOVA) followed by Dunnett's test.

**Table 3:** Effect of carnosol, ursolic acid, and oleanolic acid in locomotor activity test.

Group	Treatment	Dose (mg/kg)	Line-crossing counts (mean ± SEM)
1	Control	—	44.6 ± 6.5

2	Carnosol	10	45.4 ± 3.5
3		30	43.7 ± 5.3
4		100	36.5 ± 3.2

5	Ursolic acid	10	45.4 ± 3.5
6		30	43.3 ± 2.4
7		100	35.9 ± 4.5

8	Oleanolic acid	10	40.8 ± 2.2
9		30	42.3 ± 3.3
10		100	34.6 ± 4.1

11	Imipramine	60	40.2 ± 2.6

12	Diazepam	1	35.5 ± 3.5

All values are expressed in mean ± SEM (*n* = 6). *P* > 0.05 compared to the vehicle group. Differences between groups were analyzed by analysis of variance (one-way ANOVA) followed by Dunnett's test.

**Table 4:** Effect of carnosol, ursolic acid, and oleanolic acid on PTZ-induced seizure.

Treatment	Dose (mg/kg)	Onset of clonic-tonic convulsions (sec)	Duration of clonic-tonic convulsions (sec)	% mortality	% protection against mortality
Control	—	72.5 ± 8.5	39 ± 4.5	100%	0
Diazepam	1	197 ± 10.6^*∗∗∗*^	15.8 ± 2.6^*∗∗*^	0.00	100
Carnosol	1	77.1 ± 12.9^n.s.^	35 ± 3.5^n.s.^	66.6	33.34
Carnosol	10	117.3 ± 6.8^*∗*^	26 ± 2.6^*∗*^	33.3	66.67
Carnosol	30	158.3 ± 11.6^*∗∗*^	17.6 ± 1.8^*∗∗*^	16.3	83.33
Carnosol	100	185.8 ± 8.8^*∗∗∗*^	7.5 ± 1.1^*∗∗*^	0.00	100
Ursolic acid	1	75.1 ± 13.5^n.s.^	33 ± 3.5^n.s.^	66.6	33.34
Ursolic acid	10	120.3 ± 7.5^*∗*^	25 ± 3.6^*∗*^	33.3	66.67
Ursolic acid	30	148.5 ± 11.5^*∗∗*^	19.6 ± 2.8^*∗∗*^	16.3	83.33
Ursolic acid	100	195.8 ± 8.8^*∗∗∗*^	15.5 ± 2.5^*∗∗*^	0.00	100
Oleanolic acid	1	78.1 ± 14.5^n.s.^	34 ± 3.5^n.s.^	66.6	33.34
Oleanolic acid	10	130.3 ± 9.5^*∗*^	23 ± 2.6^*∗*^	33.3	66.67
Oleanolic acid	30	150.8 ± 13.5^*∗∗*^	18.5 ± 2.8^*∗∗*^	16.3	83.33
Oleanolic acid	100	190.8 ± 8.8^*∗∗∗*^	14.5 ± 2.5^*∗∗*^	0.00	100

The data represent the mean ± SEM (*n* = 6). ^*∗*^ *P* < 0.05, ^*∗∗*^ *P* < 0.01, and ^*∗∗∗*^ *P* < 0.001 represent significant difference compared to PTZ-induced seizure control.

n.s.: not significant.
